# Global Awareness Landscape for Ailments—A Twitter Based Microscopic View Into Thought Processes of People

**DOI:** 10.3389/fdata.2019.00018

**Published:** 2019-08-08

**Authors:** Durga Toshniwal, Soumya Somani, Rohit Aggarwal, Preeti Malik

**Affiliations:** ^1^Department of Computer Science, Indian Institute of Technology Roorkee, Roorkee, India; ^2^Department of Computer Science, Symbiosis Institute of Technology, Symbiosis International University, Pune, India; ^3^Department of Computer Science, Indian Institute of Information Technology, Allahabad, India

**Keywords:** data mining, world health, social media, epidemics, ailments, twitter data analytics

## Abstract

In this day and age, people face a lot of stress due to the fast pace of life. Due to this, people in today's digital age, suffer from a plethora of ailments. It is universally accepted that a greater awareness of ailments and their corresponding symptoms leads to an increased lifespan and better quality of life. Early detection and screening can help doctors nip diseases in their natal stages. However, not everyone is aware of them, which makes it a global issue. The study of the degree of disease awareness amongst people belonging to different nations and continents is a matter of great interest. One method that is suitable for this purpose is using clinical data. But, this data is not readily available. However, today a plethora of platforms are available to people to share their thoughts and experiences. People post about many of the important events in their lives on social media. Their posts offer a microscopic view into their lives and thought processes. Based on this intuition, twitter data pertaining to various chronic and acute diseases has been collected. Tweets for 30 deadly ailments have been collected over a period of 3 months amounting to a total of 19 million. A feature extraction approach is proposed which is used to identify the disease awareness levels across different nations. Deriving the global awareness landscape for ailments can help to identify regions which are well aware and also those that need to get aware. Clustering has been used for this purpose.

## Introduction

With the success of Web-2.0, it has become a quotidian task for web users to express their views on a myriad of issues. Web 2.0 has given an opportunity to its users so that they can interact and collaborate to create texts of their thinking and understanding on this virtual platform. This has given birth to many web applications, social-networking sites, video sharing sites, blogs, hosted services, and wikis among other things.

Consequently, many social platforms are available to people for sharing their thoughts on a variety of topics, events and products. Most of these posts chronicle their daily activities and struggles. People post about all the relevant and irrelevant events in their lives. Not all of these are useful but many of them can be used to gain an insight into society. These can be collected and the useful information can be selected by applying multiple data analytic and mining techniques.

The field of world health can vastly benefit from analyzing this data. A number of people share their health struggles and their opinions on health concerns around them on social media. Many compulsively post regular updates on the diseases that they themselves or their close relatives suffer from. People also express their concern about the diseases that are currently widespread in their localities on their social media. An analysis of these posts can be very helpful in finding a disease's spreading pattern or at the very least help us in determining disease awareness patterns among the citizens of various countries. This information can be used as a preparatory measure by the government and citizens of various countries.

Twitter has become a popular source of data in the last decade. The posts are brief, and therefore, they effectively convey a person's opinion in just a few words making it useful for the purpose of research. Twitter is also very convenient for all internet users and since the internet is ubiquitous in today's day and age, it can be called the virtual realization of all thoughts prevalent in today's society at a given point in time. Many researchers have already established Twitter as a useful source of information or data while working on many topics including public health (Sriram et al., 2010).

In this paper, ailments have been classified into chronic and acute ailments (which are to be identified differently) thereby forming two sets of the problem. Each disease also needs to be worked upon individually as all of them are different from each other in one way or another.

The rest of the paper is organized as follows: Section Literature Review gives the literature review of related topics. Section Proposed Work gives the details of the proposed work for the paper. Section Experiments and Discussion comprises of the dataset details, experiments conducted on them and the results from them. Section Conclusion and Future Work is the conclusion and future work of the report. And the references are given in the last section.

## Literature Review

In this section of the paper, some of the related research works in this field have been described briefly.

Not much work has been done to analyze twitter data for the purpose of determining awareness levels of diseases in various countries across the globe. So far research works have focused on some particular ailments or on the observations from a specific country. There is a need to perform a study that spans across a large set of common ailments in order to generate a complete picture of the awareness levels of various diseases in different countries around the world.

Research by Paul and Dredze (2011) gives an analysis of the health issues that can be studied using data from Twitter. This work focuses on the tweets collected from the United States. Results from this work show that most of the ailments that are studied can be predicted with accuracy in terms of the location of the patient, except for the deadly ones in which the patients' relatives and not the patients themselves might be tweeting. So, the tweets may be from a location which is differrent from the patients' location thereby reducing the accuracy.

There are a few studies regarding the occurrence of influenza in the United States during different years, pertaining to different kinds of work in the fields of disease pattern, location pattern etc. Influenza occurs in all the seasons with different intensity and different regions making it an interesting subject.

One approach given by Signorini et al. (2011) has combined the analysis of the occurrence of H1N1 and influenza on a weekly cross-validated dataset. The results of the prediction were cross-checked with the actual statistics of occurrence of the two diseases with an average error of 0.28% and standard deviation of 0.23%.

Another approach given by Aramaki et al. (2011) focuses on separating negative tweets that show the person not having influenza, from positive tweets which actually indicate influenza occurrence. Results show that it could successfully filter out negative tweets with f-measure = 0.76 and it detects influenza with a high correlation ratio of 0.89.

Yet another approach given by Smith et al. (2016) using Twitter data from the influenza epidemic of 2012-2013 in the United States, majorly works on distinguishing between the tweets that show awareness toward the disease and the tweets that actually show an ailment. Results from the model show that occurrence of disease has very different trends than that of its awareness. It has also shown that disease trends vary on a regional basis but awareness trends do not vary as much across different regions. Similarly, some other diseases like Dengue, HIV, H1N1, Zika etc. are also discussed using twitter data.

Based on the detailed literature survey done, it can be observed that most of the existing research works are based on the analysis of social media data from specific locations or sets of locations. Thus, there is a great scope to develop techiniques that work on data collected on a national or global level.

Also there is no centralized data on occurance patterns of diseases on a national or global level collected by the governments or other agencies.

Further, most of the research works done so far are targeted toward the analysis of a set of few ailments only. However, there is a lack of research work that holistically covers a broad spectrum of ailments.

In the present work, we aim to address some of the limitations as mentioned above.

## Proposed Work

**Figure 1:**
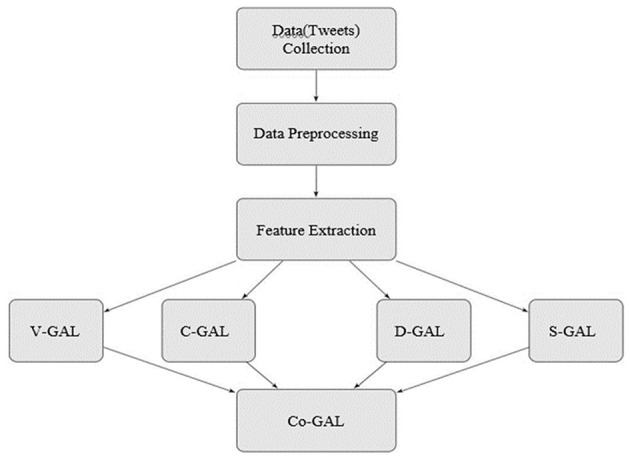
Proposed Framework, V-GAL is Visual Global Awareness Landscape, C-GAL is Continent based Global Awareness Landscape, D-GAL Disease based Global Awareness Landscape, S-GAL Similarity based Global Awareness Landscape, Co-GAL Consolidated Global Awareness Landscape.

In order to determine the awareness levels about various ailments in various countries across the globe, the following framework (as per Figure 1) has been proposed.

### Proposed Framework

To find the awareness levels using any kind of epidemiological data is not feasible as the data is not readily available due to privacy issues and the data that is available is insufficient to cover the entire globe. Thus, an approach to determine a worldwide analysis of public health awareness amongst people using twitter data has been given here.

The proposed framework for the work is illustrated in Figure 1.

#### Data Collection

The data was collected from twitter live stream using the twitter API over a period of 3 months. Twitter live stream allows us to connect to the twitter API and open a pipeline for selected data to be delivered to us. A total of 30 ailments were chosen based on the level of severity and spread. Only those ailments which were being discussed on twitter were considered. Different keywords regarding each ailment were used to collect this data from the stream. The Tweepy library was used to access the twitter API.

#### Data Biases

Only English language tweets were collected. This was done to avoid transation of non english tweets since such translation will yeild noisy data. Therefore, this work does not include any expression done by people on non english languages. There was no thresholding applied to the volume of tweets from a nation. Also, various nations of the world will have largely varying population and hence bigger countries will have more tweets. This will introduce a bias toward such countries in awareness levels. To prevent this, normalization of the number of tweets from a country with respect to its population has been done.

#### Data Preprocessing

Not all of the collected tweets have the location attribute in them. The location tagged tweets are thus separated out for further analysis. This is achieved by filtering out the tweets that had null or garbage values as their location values. The tweets are processed using Google Geo-coding API to determine the country from where the tweets are posted. The corpus of the tweets is then segmented based on their country. It is also segregated into tweets about chronic and acute ailments based on their keywords and noisy tweets i.e., tweets containing non-english words, very few words etc. are filtered out.

#### Feature Extraction

Feature Vectors are derived to give clusters of countries with similar awareness.

The feature vectors are derived as follows:

Let C be the set of countries given as per Equation 1:

(1) $$\begin{array}{l} \text{C}=\left\{{\text{C}}_{1},\ldots ,{\text{C}}_{\text{i}},\ldots ,{\text{C}}_{\text{n}}\right\} \end{array}$$

Further, let the set of chronic ailments be denoted by A_chj_:

(2) $$\begin{array}{l} {\text{A}}_{\text{chj}}=\left\{{\text{A}}_{1},\ldots ,{\text{A}}_{\text{j}},\ldots ,{\text{A}}_{\text{c}}\right\} \end{array}$$

And let the set of actue ailments be denoted by A_aj_:

(3) $$\begin{array}{l} {\text{A}}_{\text{aj}}=\left\{{\text{A}}_{\text{c}+1},\ldots ,{\text{A}}_{\text{j}},\ldots ,{\text{A}}_{\text{m}}\right\} \end{array}$$

Let the set of all ailments be denoted by A:

$$\begin{array}{l} \text{A}={\text{A}}_{\text{chj}}\text{ U }{\text{A}}_{\text{aj}} \end{array}$$

Thus,

(4) $$\begin{array}{l} \text{A}=\left\{{\text{A}}_{1},\ldots ,{\text{A}}_{\text{j}},\ldots ,{\text{A}}_{\text{m}}\right\} \end{array}$$

The corpus of tweets, T is given as per Equation 5:

(5) $$\text{T}=\sum_{\text{i}=1}^{\text{m}}{\text{T}}_{\;\text{i}}\;$$

Where T_i_ is the total number of tweets from country C_i_ given as per Equation 6:

(6) $${\text{T}}_{\text{i}}=\sum_{\text{j}=1}^{\text{m}}{\text{T}}_{\text{ij}}$$

Where,

*T*_ij_ = The number of tweets from country i about ailment j.

Let the population of country C_i_ be denoted by P_i_ and P be the world population^1^. Then:

(7) $$\text{P}=\;\sum_{\text{i}=1}^{\text{n}}{\text{P}}_{\text{i}}$$

The tweets have been segregated based on location coordinates as discussed in the Data Preprocessing section.

The proposed approach for awareness level indication using feature vectors is

Let the Feature Vector for a country C_i_ be denoted by FV_i_ as per Equation 8:

(8) $$\text{F}{\text{V}}_{\text{i}}=({\text{A}}_{1}^{\prime },\ldots ,{\text{A}}_{\text{j}}^{\prime },\ldots ,{\text{A}}_{\text{m}}^{\prime })$$

Where,

$${\text{A}}_{\text{j}}^{\prime }=({\text{T}}_{\text{ij}}/{\text{T}}_{\text{i}})$$

Feature vector of a country C_i_ for chronic diseases is called the Chronic Ferature Vector and is given as per Equation 9:

(9) $$\text{CF}{\text{V}}_{\text{i}}=({\text{A}}_{1}^{\prime },\ldots ,{\text{A}}_{\text{j}}^{\prime },\ldots ,{\text{A}}_{\text{c}}^{\prime })$$

Similarly, the Acute Feature Vector for a country C_i_ is given as per Equation 10:

(10) $$\text{AF}{\text{V}}_{\text{i}}=({\text{A}}_{\text{c}+1}^{\prime },\ldots ,{\text{A}}_{\text{j}}^{\prime },\ldots ,{\text{A}}_{\text{m}}^{\prime })$$

Thus,

(11) $$\begin{array}{l} \text{F}{\text{V}}_{\text{i}}=\text{AF}{\text{V}}_{\text{i}}\text{ U }\text{CF}{\text{V}}_{\text{i}} \end{array}$$

After the Feature Vectors are derived, Link Based, and Agglomerative Clustering methods are applied to get clusters of countries with similar awareness.

The aim of clustering is as follows:

- Given an ailment, the aim is to determine a group of countries showing similar awareness levels for it.
- Given a country, the objective is to find the top ailments being discussed.
- And lastly, we need to determine the countries that have similar top scoring ailments.

#### Visual Global Awareness Landscape (VGAL)

A Tweet Index has been defined to create the Visual Global Awareness Landscape (VGAL). It gives the level of awareness about various diseases for every country based on its normalized population. It is defined as per Equation 12.

(12) $$\begin{array}{l} \text{Tweet Index}=\left(\left({\text{T}}_{\text{i}}/{\text{P}}_{\text{i}}\right)\text{x}\left(\text{P}/\text{T}\right)\right) \end{array}$$

#### Continent Based Global Awareness Landscape (CGAL)

In this landscape, a discussion has been given regarding the diseases that people are most aware of in each continent. Acute and Chronic diseases have been discussed separately. So, the top scoring acute and chronic diseases for each continent have been determined in this landscape.

#### Disease Based Global Awareness Landscape (DGAL)

A disease based discussion has been presented regarding the countries that have the most awareness about each disease. Also, the top scoring diseases being discussed in each country are compared to the most prevalent ailment in that country. Acute and Chronic diseases have been considered separately for this purpose.

#### Similarity Based Global Awareness Landscape (SGAL)

In this landscape, clustering algorithms have been applied on CFV and AFV sets to determine similarity based groups of countries. Clusters of countries are formed such that within a cluster, similar awareness levels exist for a common set of diseases. Two methods of clustering which are inspired by Guha et al. (2000) and Kaufman and Rousseeuw (1990) have been applied to the CFV and AFV sets. The methods are: Link Based and Agglomerative Clustering.

#### Consolidated Global Awareness Landscape (Co-GAL)

The CVF and AVF sets have further been analyzed to give the Consolidated Global Awareness Landscape which comprises of:

- Holistic Awareness Profile (HAP): This consists of countries that have awareness about all the ailments considered. Such countries are not in immediate need of awareness campaigns for diseases. The can also mentor other countries to help them in becoming more aware against diseases.
- Specific Awareness Profile (SAP): It consists of countries that have awareness about some specific ailments.
- Negligible Awareness Profile (NAP): This consists of countries that have the least awareness. These countires are in immediate need of awareness campaigns against various diseases.

Lastly, geographical aspects have been considered to deternmine the geographical closeness of countries lying in the same cluter. Also, the actual occurance of ailments has been considered to determine the correlation between the occurance and awareness levels of ailments.

## Experiments and Discussion

The following section contains the dataset description that gives us the total number of ailments considered in this work along with the number of acute and chronic diseases. This section also presents the results obtained in this work.

### Dataset Description

The data was collected from twitter live stream using twitter API over a period of 3 months. As per Tables 1, 2, 30 ailments in total were chosen based on the level of severity and spread.

**Table 1:** Dataset description.

**Sr. no**.	**Attributes**	**Values**
1	No. of chronic diseases	10
2	No. of acute diseases	20
3	Duration	July-Sept 2017
4	Total tweets collected	19,301,623
5	Total countries	244

**Table 2:** Ailment description.

**Sr. no**.	**Type of**	**No. of**	**Namely**
	**ailment**	**ailments**	
1	Chronic	10	Cancer, chikungunya, diabetes, heart diseases, hepatitis, HIV, leprosy, RHD, TB and toxoplasmosis
2	Acute	20	Chickenpox, cholera, dengue, diarrhea, ebola, H1N1, influenza, Japanese encephalitis, lassa fever, malaria, measles, mumps, pertussis, rift valley fever, Smallpox, syphilis, typhoid, typhus, yellow fever, zika

Ailments have been classified into two categories: Chronic and Acute. An ailment that develops over a longer period of time and lasts for more than a period of 3 months is known as a chronic ailment and an ailment that comes rapidly and lasts for a short period of time is categorized as an acute ailment.

### Discussion

After the data has been processed and the various steps specified in the proposed framework have been carried out, the following results have been obtained. These results give us a holistic picture of the global awareness levels of various ailments.

#### Visual Global Awareness Lanscape (VGAL)

**Figure 2:**
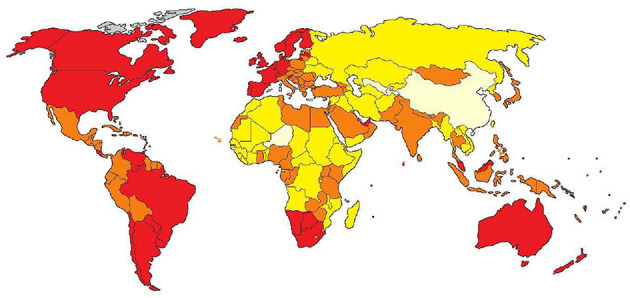
Visual Global Awareness Landscape.

Figure 2 shows the awareness levels of each country based on the normalized tweets per person (given by the Tweet Index). The most aware countries are represented in red and the least aware are represented in light yellow. The awareness for each color is:

*Red* = *1; 0.2* ≤ *orange *<* 1; 0.1* ≤ *yellow *<* 0.2 and 0* ≤ *light yellow *< *0.1*

Red denotes high awareness, orange denotes medium awareness, yellow denotes low awareness, and light yellow represents the least aware countries.

#### Continent Based Global Awareness Lanscape (CGAL)

Based on location of generation of the tweets, they can be divided amongst the seven continents. The statistics for each of the continents can be seen in Table 3. The % Column depicts the percentage of tweets from the continent with respect to the world. The top chronic column gives the top scoring chronic ailment for that particular continent. Similarly, the top acute column gives the top scoring acute ailment for that continent.

**Table 3:** Continent based statistics based on twitter data collected.

**Sr. no**.	**Continent**	**% of tweets**	**Top chronic**	**Top acute**
1	North America	51.1	Cancer	Zika
2	Europe	16.52	Cancer	Cholera
3	South America	14.88	TB	Cholera
4	Asia	11.13	Cancer	Cholera
5	Africa	4.73	Cancer	Cholera
6	Oceania	1.61	Cancer	Cholera
7	Antarctica	0.01	Cancer	Dengue, cholera

Out of the chronic diseases, cancer is prevalent in all of the continents except for South America. Tuberculosis (TB) is the most prevalent chronic ailment in South America. This can be explained by the fact that Brazil has a high occurrence of TB and most of the tweets from South America (around 73%) are from Brazil.

Out of the set of the acute ailments, Cholera, Dengue and Zika have the most awareness in various continents (refer Table 3).

#### Disease Based Global Awareness Lanscape (DGAL)

All the ailments have been classified into Acute and Chronic ailmets. The top scoring ailments from each category are given in Tables 4, 5. The % column in the Tables 4, 5 give the percentage of tweets for each disease with respect to the total number of tweets from the world. Top scoring countries column gives the countries that have the highest number of tweets for a given ailment. The most prevalent ailment as per Tables 4, 5 signify the most commonly occuring ailment in that specific country.

**Table 4:** Top chronic ailments and the countries that majorly discuss about them.

**Ailment**	**% of tweets**	**Top scoring countries (%)**		**Most prevalent ailment**
Cancer	45	Unites States	60	Cancer (for all)
		United Kingdom	9	
		France	3.56	
		India	2.95	
		Canada	2.79	
TB	26	Brazil	40	TB for Brazil, Spain, Portugal and cancer for UK and US
		Unites States	22.5	
		Spain	4.36	
		Portugal	4.22	
		United Kingdom	3.25	
HIV	11.7	Unites States	45.6	Cancer (for all)
		United Kingdom	9.62	
		South Africa	5.89	
		Nigeria	3.78	
		India	3.41	
Diabetes	10.7	United States	47	Cancer for UK, US, India, TB for Brazil and diabetes for Indonesia
		United Kingdom	9.35	
		Brazil	5.96	
		Indonesia	3.78	
		India	3.5	

**Table 5:** Top Acute Ailments and the countries that majorly discuss about them.

**Ailment**	**% of tweets**	**Top scoring countries (%)**		**Most prevalent ailment**
Dengue	1.85	India	28	H1N1 in India, dengue in Pakistan and Mexico, cholera in US and zika in Brazil.
		Pakistan	14.6	
		United States	13	
		Brazil	6.64	
		Mexico	5.21	
Zika	1.77	United States	40	Dengue in US and Mexico, zika in Brazil and Venezuela, H1N1 in India
		Brazil	26	
		India	4.36	
		Mexico	3.66	
		Venezuela	2.95	
Cholera	1.66	United States	31.6	Cholera for all
		Kenya	12.16	
		United Kingdom	11.18	
		Poland	6.62	
		Nigeria	3.91	
Measles	0.8	United States	40	Cholera in US, UK and Australia, measels in Indonesia and H1N1 in India
		Indonesia	16.4	
		United Kingdom	9.85	
		India	6.08	
		Australia	3.28	

#### Top Chronic Ailments

Cancer has the highest % of tweets among all the chronic ailments, making it the most talked about disease all over the world. Other top scoring chronic ailments are TB, HIV and Diabetes.

Table 4 gives the top chronic ailments along with the top scoring countries for each ailment. For example, Brazil, Spain and Portugal have the maximum number of tweets about TB making them areas of high concern of TB. The most prevalent chronic ailments in the top scoring countries have also been given in Table 4.

#### Top Acute Ailments

Table 5 gives the top scoring acute ailments along with the top scoring countries for each ailment and the most prevalent acute ailments in those countries.

Out of all the countries discussing about dengue, only Pakistan and Mexico have it as the most prevalent acute disease.

However, all of the five countries most concerned about cholera have it as their most prevalent acute ailment.

#### Similarity Based Global Awareness Lanscape (SGAL)

To determine a similarity based global awareness landscape, clustering has been done on the set of country wise Feature Vectors, FV. Acute and chronic ailments have been considered separately for this landscape.

A total of 22 clusters of countries having similar awareness levels for chronic diseases have been generated. The major results have been presented in Table 6. It gives the size of the cluster, some of the important countries in that cluster and the similarity traits for that cluster.

**Table 6:** Clusters of countries for chronic ailments.

**Cluster No**.	**No. of countries**	**Countries**	**Ailment(s)**
1	56	Algeria, Fiji, Greece, Canada, Australia, United Kingdom, United States, Nigeria, India etc.	Cancer 50–70%
2	47	Afghanistan, Kazakhstan, Japan, Hong Kong, China, Tajikistan, Hungary, Romania etc.	Cancer and TB 35–40%
3	19	Switzerland, Denmark, Nepal, Bangladesh, Sri Lanka, Vietnam etc.	Cancer, TB, HIV and diabetes 10–40%

For acute ailments, 38 clusters have been generated. They have sizes ranging from 2 to 12. The clusters consist of groups of countries that have similar levels of awareness for acute ailments. The most important results have been presented in Table 7. For example, Albania, Mauritiana, North Korea etc., have similar awareness for cholera and ebola.

**Table 7:** Clusters of countries for acute ailments.

**Cluster No**.	**No. of countries**	**Countries**	**Ailment(s)**
1	8	Albania, Mauritiana, North Korea, Montenegro, Norway, Tunisia etc.	Cholera, ebola
2	7	Algeria, Turket, Samoa, Vanuatu, India, Myanmar (Burma) etc.	Dengue, cholera
3	5	Afgahnistan, Djibouti, Pitcairn Islands, Cook Islands and Equitorial Guinea	Cholera

This landscape gives similarity based groups of countries, which are clusters of countries that have similar awareness levels about similar diseases.

When the feature vectors for ailments are compared, a few of them show very similar awareness spread. Syphilis, Pertussis and Small Pox have a similar spread in terms of the number of tweets. Similarly, the awareness spread for HIV and Ebola, Chickenpox and Mumps are quite similar.

#### Consolidated Global Awareness Lanscape (Co-GAL)

Highly aware countries are countries that have awareness about all the considered ailments i.e. countries having citizens tweeting about all the considered ailments. Only seven countries, namely Australia, Canada, France, India, Thailand, UK and US, are highly aware countries these can be classified into HAP. Countries like Argentina, Brazil, Nigeria etc lack in awareness of some ailments despite having a large number of total tweets. These are classified into SAP. Such countries must not be mistaken for highly aware countries since they lack in awareness about some of the considered ailments.

The awareness and actual occurrence of ailments can be compared and the countries can be divided into four groups based on this comparision. The groups are as follows:

- Countries with both occurrence and awareness.
- Countries that have awareness but no occurrence.
- Countries that have occurrence but no awareness.
- Countries that have neither occurrence nor awareness.

This has been illustrated in Table 8.

**Table 8:** Incidence and awareness comparision.

	**Countries having awareness (HA)**	**Countries not having awareness (NA)**
Countries having incidence (HI)	Countries having incidence and awareness of ailments (HIHA)	Countries having incidence but no awareness about ailments (HINA)
Countries not having incidence (NI)	Countries not having incidence but having awareness about ailments (NIHA)	Countries neither having incidence nor awareness about ailments (NINA)

As an example, consider Table 9 which gives the occurrence and awareness comparision for TB in various countries of the world. Table 9 gives us various countries that have both awareness and occurrence of TB and also countries that have neither.

**Table 9:** Incidence and awareness comparision for TB.

	**Countries discussing about TB**	**Countries not discussing about TB**
Countries under high TB burden^a^	Brazil, India, Philippines	Indonesia, China, Nigeria, Pakistan, South Africa, Bangladesh, DR Congo, Ethiopia, Myanmar, UR Tanzania, Mozambique, Vietnam, Russian Federation, Thailand, Kenya, Uganda, Afghanistan, Cambodia and Zimbabwe
Countries not under TB burden^a^	US, Spain, Portugal, UK, Argentina, Chile and France	Rest of the world

a *www.who.int/tb/publications/global_report/en/*.

## Conclusion and Future Work

In the present work, data has been collected from a twitter live stream. A set of analytics and processing has been applied to the collected data to determine the awareness levels in each country or continent regarding each ailment. An approach for feature extraction has been proposed. The feature vectors hence derived are used for clustering. The primary aim of clustering is to determine clusters of countries with similar awareness levels. Various aspects namely, Visual Global Awareness Landscape (VGAL), Continent based Global Awareness Landscape (CGAL), Disease based Global Awareness Landscape (DGAL), Similarity based Global Awareness Landscape (SGAL), and Consolidated Global Awareness Landscape (Co - GAL), have been determined to present a holistic picture of the global awareness landscape of various ailments. This work has revealed that discussion or awareness about ailments and their incidence is not necessarily co-occurring. The analysis has also revealed that the countries can be divided into four groups namely:

- Countries having incidence and awareness of ailments.
- Countries not having incidence and awareness of ailments.
- Countries having incidence and no awareness of ailments.
- Countries niether having incidence and nor awareness of ailments.

The results of this work can be used by the governments of various nations and also international agencies like WHO to determine the countries that need immediate awareness drives for various diseases. Also, the nations that are highly aware can mentor other nations to spread awareness about these ailments. There is no centralized repository of global data available hence a direct comparitive study may not be possible. In the present work emphasis is placed on spatial analysis. A temporal analysis can also be done, which can also be seen as the future scope of the work.

## Data Availability

The datasets for this study will not be made publicly available because The datasets are a part of sponsored research project and therefore cannot be made available directly in form of open data.

## Author Contributions

DT: conceptualization of the proposed methodology, idea, and guidance. SS, RA, and PM: partial implementation and documentation.

### Conflict of Interest Statement

The authors declare that the research was conducted in the absence of any commercial or financial relationships that could be construed as a potential conflict of interest.

## Footnotes

^1^https://en.wikipedia.org/wiki/List_of_countries_by_population_(United_Nations)
